# Reduction of Total Phenols in Virgin Olive Oil as a Preservation Medium during Cold Storage of Whey Cheese and Tofu

**DOI:** 10.17113/ftb.62.03.24.8434

**Published:** 2024-09

**Authors:** Valerija Majetić Germek, Ivana Gobin, Doris Franjković, Marija Marković, Olivera Koprivnjak

**Affiliations:** 1 University of Rijeka, Faculty of Medicine, Department of Food Technology and Quality Control, Braće Branchetta 20, 51000 Rijeka, Croatia; 2 University of Rijeka, Faculty of Medicine, Department of Microbiology and Parasitology, Braće Branchetta 20, 51000 Rijeka, Croatia; 3 University of Rijeka, Faculty of Medicine, Braće Branchetta 20, 51000 Rijeka, Croatia

**Keywords:** virgin olive oil, whey cheese, tofu, total phenols, Fast Blue BB test

## Abstract

**Research background:**

Virgin olive oil, known as a good source of health-promoting hydrophilic phenols, is traditionally used as a medium for preserving various foods. Phenols in general can form complexes with proteins, but there is little information on the direct contact of virgin olive oil with protein-rich food during long-term storage. In this study, the dynamics of the decline of total phenols in oil used as preservation medium for a traditional (whey cheese skuta) and a modern product (tofu) were compared.

**Experimental approach:**

Pieces of skuta or tofu immersed in virgin olive oil at different food-to-oil mass ratios were stored in the refrigerator for up to 21 days. The oil quality indices, water content and the total count of aerobic mesophilic bacteria in the immersed materials were monitored. To determine the total phenols in the oil, the optimal conditions of the Fast Blue BB test, which is a suitable alternative to the standard method, were tested and selected.

**Results and conclusions:**

The effect of both materials on the indicators of hydrolytic and oxidative deterioration of the oil is almost identical (a gradual decrease), which is most likely due to the continuous release of water from the immersed food. A sharp decrease in total phenols in the oil (by about 50 %) after seven days of storage in contact with both materials indicates a combination of causes (water-to-oil migration and phenol-protein interactions). The form of the rational function is highly representative of the decrease in total phenols during the first seven days of tofu/oil storage, indicating a very rapid interaction with tofu proteins. The preservative effect of virgin olive oil in terms of microbiological spoilage was not observed.

**Novelty and scientific contribution:**

The results of this study contribute to the knowledge on the dynamics of phenol-protein interactions and emphasise the need for further investigations on traditional or newly used protein-rich foods preserved in direct contact with virgin olive oil, taking into account possible changes in the functional, nutritional and sensory properties of phenols and proteins.

## INTRODUCTION

Skuta is a traditional name for the whey cheese produced in Croatia, which is obtained by exposing the whey to heat and acid and then draining it moderately for up to two days. It belongs to the soft cheese category (water content >55 %) (*1*) and has a texture that allows it to be cut into pieces. Due to its low salt content, its relatively high pH value (around 6.5) and unsuitability for thermal pasteurisation after packaging, the usual shelf life of skuta is only 6 to 7 days at 4 to 8 °C (*2*). To extend this period, the cut pieces of skuta are sometimes dipped in virgin olive oil and preserved. The preserving effect of virgin olive oil as a liquid medium can be attributed to the limited access of air, the content of hydrophilic and lipophilic phenols and the favourable composition of fatty acids in terms of oxidative stability. A similar practise is used in the preservation of some other types of cheese, vegetables and fish, where migrations of ingredients and their interactions may occur (*3*-*6*). It is known that phenols in general can form complexes with proteins, leading to reciprocal changes in structural, functional, nutritional and sensory properties (*7*, *8*). Such changes at the molecular level depend on the chemical structure of both phenols and proteins, their mass ratio and the properties of the medium in which they react. Phenols extracted from olive fruit have attracted considerable attention due to the characteristic sensory properties they confer to virgin olive oil (bitterness and pungency) and their bioactivity in alleviating various chronic diseases (*9*). The changes induced by the contact of total or individual phenols from olive fruits with proteins were mainly studied in protein-rich foods to which phenolic extracts were incorporated as an ingredient (*10*-*13*) or which (such as pasta filata cheese) were immersed in aqueous solutions with added phenolic extract (*14*). As far as we are aware, there are only a few studies that consider changes caused by direct contact of virgin olive oil with protein-rich food during long-term storage, *e.g.* pieces of tuna (*6*) and various cheeses (*4*). In all these studies, a statistically significant reduction in the mass fraction of olive fruit-derived phenols was observed during product storage, which could be partly due to their antioxidant activity and partly due to interactions with proteins or other components of the food. The effect of virgin olive oil on the growth of microorganisms during storage of dairy products immersed in this medium has also been little studied. As far as we know, there are only a few reports on this subject relating to the preservation of mature cheese in olive oil (*15*) and of dried yoghurt cheese (labneh anbaris) in virgin olive oil and some refined vegetable oils (*16*, *17*).

Tofu, a well-known soy product, is similar in texture and culinary use to soft cheese. Therefore, cut pieces of tofu immersed in extra virgin olive oil could be interesting as a new product option with improved organoleptic and nutritional properties. Due to the similar texture and content of macronutrients, the effect of tofu on the phenols in extra virgin olive oil could be similar to that of skuta. However, the differences in the structure and amino acid composition of soy and whey proteins could lead to different types and extent of interactions with virgin olive oil phenols. Therefore, the aim of this study is to compare the dynamics of the decline of total phenols in the oil used as a preservation medium for a traditional (whey cheese in virgin olive oil) and a modern product (tofu in virgin olive oil) in terms of their macronutrient characteristics and water migration into the oil.

For the determination of total and individual phenolic substances in virgin olive oil, the International Olive Council (IOC) has adopted the HPLC methods (*18*) after extraction with an aqueous alcoholic solution or after solid phase extraction. For the routine determination of total phenols, the Folin-Ciocalteu colorimetric method applied to an aqueous alcoholic extract is widely used. The main disadvantage of the Folin-Ciocalteu method is the insufficient accuracy and low specificity due to numerous interfering substances from the extract (*19*). Recently, Siano *et al.* (*20*) proposed a simple colorimetric method that has higher selectivity and sensitivity than the Folin-Ciocalteu method and correlates strongly with the IOC HPLC-UV method using the aqueous alcoholic extract. The method is based on the reaction of the Fast Blue BB diazonium salt with hydroxyl groups on the aromatic rings of the phenols in alkaline medium, followed by the spectrophotometric measurement of the stable yellow colour formed at 420 nm. As the reaction is performed directly in virgin olive oil, sample manipulation, possible degradation of phenolic compounds during extraction or incomplete extraction are minimised (*19*, *20*). Therefore, such a simple, fast, cheap and reliable alternative to the commonly used methods for the determination of total phenols could be very interesting to monitor changes in bioactive phenols of virgin olive oil under different production conditions, such as during prolonged contact with different immersed foods. In order to select the optimal conditions for the Fast Blue BB test in the case of immersed skuta or tofu, certain combinations of emulsification techniques and reaction times were tested and compared with respect to phenol yield.

## MATERIALS AND METHODS

### Materials

Commercial cow’s whey cheese, *i.e.* skuta (Mini Dairy Veronika, Desinić, Croatia) was purchased at the local retail shop, while tofu (Vemondo, Austria) was purchased at the local supermarket (Rijeka, Croatia). According to the average nutritional information on the label, skuta contained 5.9 g fat (of which 4.1 g saturated fat), 3.5 g carbohydrates (of which 3.5 g sugar), 8.6 g protein and 0.5 g salt. Tofu contained 7.1 g fat (of which 1.2 g saturated fat), 3.7 g carbohydrates (of which 0.5 sugar), 12 g protein and 0 g salt. The extra virgin olive oils produced in crop year 2022/23 were purchased from Family Agricultural Holding Bellé Ervin (Buje, Croatia). Fast Blue BB hemi (zinc chloride) salt with a dye content ≥80 % was purchased from Sigma-Aldrich, Merck (Steinheim, Germany). Ethanol 96 % (UV-IR-HPLC grade) and caffeic acid (purity 99 %) were purchased from Panreac (Barcelona, Spain).

### Sample preparation

Transparent glass jars (170 mL) with threaded metal caps and metal and glass utensils were sterilised at 180 °C for 60 min and then cooled at room temperature. Skuta and tofu were evenly cut into small cubes (1.5 cm×1.0 cm×1.0 cm). Approximately 97 g of skuta or tofu was placed in a sterilised jar, completely filled with virgin olive oil (75 g on average) to completely cover the cubes, sealed with metal caps and stored in a refrigerator at (2±2) °C for 7, 14 or 21 days. Samples of skuta or tofu in virgin olive oil in a mass ratio of 1.3, *i.e.* 44 % oil in the food/oil mixture were prepared in triplicate for each time point of the experiment, while three jars filled to the brim with virgin olive oil served as control samples. Prior to the chemical analyses, the contents of the jars were homogenised by gentle shaking. The control virgin olive oil and the virgin olive oil separated from the skuta or tofu were filtered through a cotton layer.

In the second part of the experiment, the jars were completely filled with tofu cubes and virgin olive oil in a mass ratio of 0.2, 0.6 and 1.3 (*i.e.* 82, 63 and 44 % oil in the tofu/oil mixture, respectively). Samples were prepared in triplicate for each tofu/oil mass ratio, while two jars filled to the brim with virgin olive oil served as control samples. The samples were stored in the refrigerator at (6±2) °C for seven days. An aliquot of the oil taken from the centre of the jar was removed each day during cold storage, filtered through a cotton layer and used for the determination of total phenols.

### Determination of virgin olive oil quality indices and water content

The free fatty acids and the spectrophotometric indices (K_232_ and K_268_) of the virgin olive oil samples were determined according to the official methods specified in the European Commission Regulation (*21*). ISO method 662:2016 (*22*) was used to measure the water content in the oil samples. The analytical procedures were carried out in duplicate.

### Fast Blue BB test for the determination of total phenols

The Fast Blue BB test for the determination of phenols was performed directly on extra virgin olive oil samples according to the method described by Siano *et al.* (*20*) with minor modifications. To an oil sample (0.70 g) weighed in a 15-mL plastic tube, 2 mL of freshly prepared ethanol solution of Fast Blue BB salt (0.1 *m*/*V*) and 2 mL of NaOH (5 % *m*/*V*) were added. Three emulsification techniques were tested on the virgin olive oil/reagent mixture in test tubes: a horizontal shaker (KS 130 Basic; Ika-Werke, Staufen, Germany), a vortex device with test tube holder (Genius 3; Ika-Werke) and an ultrasonic bath (140/560 W, 35 kHz; Bandelin Sonorex Digitec, Berlin, Germany) were used. The tested reaction time was 10, 20 and 30 min. The contents of each test tube were briefly homogenised (2 min) on a vortex device before horizontal shaking and sonication. After emulsification, the test tubes were centrifuged (centrifuge model EBA 200; Hettich, Tuttlingen, Germany) at 3461×*g* for 5 min. The separated hydro-alcoholic layer was transferred to a new test tube and also centrifuged at 3461×*g* for 5 min. The absorbance was measured at 420 nm in optical glass cuvettes using a UV-Vis spectrophotometer (model DR/400; HACH, Loveland, CO, USA). A calibration curve was prepared with the standard caffeic acid solution in ethanol (1 mg/mL), which was pipetted from 150 to 700 µL to nine pear-shaped flasks and evaporated on a rotary evaporator (RV 10 digital; Ika-Werke). The reagents for the Fast Blue BB test were added to the dry residues of caffeic acid and shaken vigorously on a vortex device (2 min) and then on a horizontal shaker (20 min). The analytical determination for each variant was repeated seven times. Total phenols are expressed in mg of caffeic acid equivalents (CAE) per kg of oil.

For the Fast Blue BB test of virgin olive oil samples used for dipping skuta and tofu, 20 min of vigorous shaking on a vortex device with a test tube holder was used as the most appropriate emulsification procedure and the analytical determination was repeated at least twice for each sample replicate.

### Microbiological analyses

Classical microbiological methods were used to determine the total aerobic plate counts and to isolate and identify pathogenic bacteria (on day 0 and after 21 days of storage). A mass of 1 g (or 25 g in 225 mL for the detection of pathogenic bacteria) of each sample was homogenised in 9 mL of sterile buffered peptone water (Biolife, Milan, Italy) for 3 min using sterile glass beads and serially diluted before plating. For total aerobic plate count, appropriate dilutions were spread plated in triplicate on nutrient agar (Biolife) and incubated at (35±2) °C for 72 h. *Salmonella* sp. was grown in Rappaport-Vassiliadis (RV) *Salmonella* enrichment broth (Merck, Darmstadt, Germany) and then subcultured on xylose lysine deoxycholate (XLD) agar (Biolife) at 37 °C for 24–48 h (*23*). *Listeria monocytogenes* was grown in Fraser broth with selective supplement (Biolife, Milan, Italy), followed by subculturing on agar *Listeria* according to Ottavani & Agosti (ALOA) with selective supplement (Biolife) at (35±2) °C for 24 h, according to ISO 11290-1:2017 (*24*). Microbial growth was determined by traditional plate counting and the results are expressed as the logarithm of colony forming units per gramme of cheese (CFU/g).

### Statistical analyses

The results were subjected to one-way ANOVA or repeated measures ANOVA to determine statistically significant differences (p<0.05) among the emulsification techniques used for the Fast Blue BB test, the days of storage of skuta or tofu in virgin olive oil and the mass fraction of oil in the tofu/oil mixture. Homogeneity of variance was tested using Levene’s test and mean values were compared using Tukey’s honestly significant difference test for equal or unequal *N*. Pearson’s linear correlation was used to relate the total phenolic content and the mass fraction of oil in the tofu/oil mixture. The Statistica v. 14.0.0.15 software (*25*) was used for the statistical analyses.

## RESULTS AND DISCUSSION

Given the high initial water content of skuta (65 %) and tofu (71 %), an increase in the water content of virgin olive oil in contact with these two materials was to be expected, although significantly higher values (three to ten times higher than the control) were only observed at the end of the three-week storage (Table 1 (*26*)). According to Papadimitrou *et al.* (*27*) and Cayuela-Sánchez and Caballero-Guerrero (*28*), the water mass fraction in virgin olive oils is on average 0.5 % and can reach up to 1.5 %, depending on the method used for oil extraction and clarification. The extra virgin olive oil used in the experiment is therefore characterised by a very low water content (≤0.07 %), probably in the form of micro-dispersed water particles. In contrast, the migration of water from the immersed material resulted in water inclusions of a transient nature (at the time of extracting an aliquot of oil for the analysis, these inclusions were visible to a lesser extent within the oil matrix and to a greater extent at the bottom of the jars). Although excess water can promote hydrolytic degradation during oil storage, this is not evident from the free fatty acid (FFA) mass fractions shown in Table 1. While the values for this indicator of hydrolytic deterioration remained stable in the control oil, they decreased significantly in the oils with both submerged materials during storage. Klisović *et al.* (*4*) reported a similar increase in moisture content but an opposite trend in the FFA of extra virgin olive oil used as a medium for the one-month preservation of skuta at 4 °C. On the other hand, the indicators of oxidative deterioration (K232 and K268) decreased statistically significantly both in this study and in the study conducted by Klisović *et al.* (*4*). According to the hypothesis proposed by Cayuela-Sánchez and Caballero-Guerrero (*28*), free fatty acids are amphiphilic molecules that can be located at the oil/water interface or, according to Lercker *et al.* (*29*), bind to particles dispersed in the oil and precipitate with them. In addition, the products of the oxidation of free fatty acids are polar substances and can dissolve in the above-mentioned water inclusions in the oil. Therefore, the observed gradual decrease in the indicators of hydrolytic and oxidative deterioration could be the result of their removal by the continuous release of water in the form of inclusions from skuta or tofu until equilibrium is reached.

**Table 1:** Mass fractions of water and total phenols in virgin olive oil used as a medium for the preservation of skuta or tofu during storage at (2±2) °C and selected quality parameters

*t*(storage)/day	*w*(water)/%	FFA as *w*(oleic acid)/%	K232	K268	*w*(total phenols as CAE)/(mg/kg)
Control					
0	(0.074±0.005)^c^	(0.15±0.00)^a^	(1.90±0.00)^ab^	(0.16±0.01)^a^	(806±8)^b^
7	(0.053±0.003)^c^	(0.15±0.01)^a^	(1.87±0.01)^ab^	(0.15±0.00)^a^	(882±66)^ab^
14	(0.059±0.009)^c^	(0.15±0.00)^a^	(1.94±0.02)^a^	(0.15±0.00)^a^	(899±69)^a^
21	(0.049±0.007)^c^	(0.15±0.01)^a^	(1.82±0.04)^b^	(0.14±0.00)^b^	(855±61)^ab^
With skuta					
7	(0.069±0.009)^c^	(0.13±0.00)^b^	(1.56±0.05)^c^	(0.11±0.00)^c^	(457±39)^c^
14	(0.12±0.03)^c^	(0.12±0.01)^c^	(1.55±0.02)^cd^	(0.10±0.00)^d^	(315±22)^d^
21	(0.23±0.04)^b^	(0.10±0.01)^d^	(1.40±0.02)^e^	(0.09±0.00)^de^	(307±26)^d^
With tofu					
7	(0.088±0.008)^c^	(0.10±0.00)^d^	(1.51±0.02)^d^	(0.10±0.00)^d^	(416±18)^c^
14	(0.08±0.01)^c^	(0.09±0.00)^e^	(1.44±0.04)^e^	(0.09±0.00)^d^	(318±11)^d^
21	(0.67±0.07)^a^	(0.08±0.01)^e^	(1.45±0.01)^e^	(0.09±0.00)^e^	(241±16)^e^
Actual limits for extra virgin olive oil category (*26*)					
	/	≤0.80	≤2.50	≤0.22	/

The results are given as mean value±standard deviation, *N*=3 for the control and *N*=6 for the addition of skuta or tofu (3 jars determined twice). Mean values within a single column labelled with different letters are statistically different (one-way ANOVA, Tukey’s test for unequal *N*, p<0.05). FFA=free fatty acids, CAE=caffeic acid equivalents, K232 and K268=indicators of oxidative deterioration, /=not applicable

As far as the microbiological aspect of product deterioration is concerned, the data in Table 2 show that the initial sample of skuta differs considerably from that of virgin olive oil and tofu. The initial

**Table 2:** Total count of mesophilic bacteria of virgin olive oil, skuta and tofu samples at the beginning and after 21 days of storage at (2±2) °C

		*t*(storage)/day	
Product	Sample	0	21
		*N*/(CFU/g)	
Virgin olive oil	Control	<1	<10
	Contact with skuta		<10
	Contact with tofu		<10
Skuta	Control	3.8·10^7^	1.2·10^8^
	Contact with oil		1.6·10^8^
Tofu	Control	<10	<10
	Contact with oil		<10

CFU=colony forming units

microbiological load of the skuta sample was quite high (7 log CFU/g) compared to 3–5 log CFU/g from other reports (*30*, *31*), and it increased by about 1 log by the end of refrigerated storage (both for the control and the sample immersed in oil). In contrast, Rao *et al.* (*16*) and Keceli *et al.* (*17*) found a decrease in the total number of aerobic bacteria, lactic acid bacteria and yeasts within 30 days of storage of labneh in oil (the product most similar to skuta in oil) at room temperature. In these two papers, the anaerobic conditions were emphasised as the most important for such an inhibitory effect of virgin olive oil, especially because no significant differences were found with vegetable oils from which phenolic compounds were removed by refining (*17*). In all virgin olive oil and tofu samples in our study, no changes were observed compared to the initial value of the microbiological load (<10 CFU/g). The values for skuta and tofu stored without immersion in oil did not differ from those of the immersed samples, although the oil used belonged to the ‘high phenols’ category. This leads to the conclusion that the preservative effect of virgin olive oil in direct contact with protein-rich foods during cold storage is not relevant with regard to microbiological spoilage. Nevertheless, it should be mentioned that the foodborne pathogens *Salmonella* spp. and *L. monocytogenes* were not present in any of the samples (data not shown) and it can therefore be assumed that all samples fulfil the microbiological criteria for foodstuffs on the EU market (*32*).

A clear effect on the phenol yield was observed with the techniques used for stirring the mixture of virgin olive oil and reagent in the Fast Blue BB test (Table 3). For this part of the study, an oil sample with the usual phenolic content for commercially available virgin olive oils (*33*) was selected. The lowest phenolic mass fraction, expressed as caffeic acid equivalents (CAE), was obtained by horizontal shaking for 10 min (338 mg/kg), while a statistically significant and linear increase was obtained when the reaction time was extended to 30 min. Vortexing for 20 min was more effective than for 10 min (364 *vs*. 344.6 mg/kg), while this effect remained the same after 30 min. Siano *et al.* (*20*) suggested the possibility of shortening the reaction time by immersing the oil/reagent mixture in an ultrasonic bath. This was confirmed by our research, so that 10 and 20 min of sonication gave a significantly higher value than horizontal shaking or vortexing for the same duration. In addition, 5 min of sonication resulted in a value comparable to 20 min of horizontal shaking and vortexing ((353±25) mg/kg, mean of seven replicates, data not shown). However, treatment in an ultrasonic bath resulted in a very cloudy hydroalcoholic phase of the reaction mixture, which in some cases required additional centrifugation to achieve complete clarity and suitability for subsequent spectrophotometric measurement. Variations in the clarity of the hydroalcoholic phase could be the reason for the higher variability of results when using ultrasound than the other two stirring methods. Although the reaction is promoted by ultrasound, due to the observed disadvantages, 20-minute vortexing was chosen as a time-saving and easy-to-perform method for the Fast Blue BB test of virgin olive oil for the preservation of skuta and tofu.

**Table 3:** Mass fraction of total phenols in virgin olive oil determined by the Fast Blue BB test using different combinations of emulsification technique and reaction time

	*t*(reaction)/min		
Emulsification technique	10	20	30
	*w*(total phenols as CAE)/(mg/kg)		
Horizontal shaker	(338±14)^bC^	(355.1±9.9)^bB^	(374.5±7.4)^abA^
Vortex apparatus	(344.6±4.8)^bB^	(364±12)^bA^	(366±17)^bA^
Ultrasonic bath	(408±16)^aAB^	(420±16)^aA^	(392±15)^aB^

The results are given as mean value±standard deviation, *N*=7. Different lowercase letters in the same column indicate significant differences (one-way ANOVA, Tukey’s test, p<0.05) among emulsification techniques, while different uppercase letters in the same row indicate significant differences (one-way ANOVA, Tukey’s test, p<0.05) among reaction times within the individual emulsification techniques. CAE=caffeic acid equivalents

The extra virgin olive oil used for the skuta and tofu preservation trials had an average total phenolic content, expressed as CAE, of 860 mg/kg and could be labelled with the term ‘high phenolic olive oil’ according to Diamantakos *et al.* (*33*), who suggested a limit of >500 mg/kg for such a claim. During the three-week storage in the refrigerator at (2±2) °C, this value remained largely constant for the control oil, *i.e.* without statistically significant deviations. In the oil samples with the addition of skuta or tofu, a significant decrease was observed after just 7 days of storage. Under the influence of skuta, the phenol mass fraction in the oil decreased by 47 % and under the influence of tofu by 52 % compared to the control. In a similar study (long-term contact of skuta with virgin olive oil at 4 °C), Klisović *et al.* (*4*) also found a decrease in the total hydrophilic phenols in the oil after one month. In their case, this decrease was less pronounced (by 35 % compared to the control) despite a longer storage time (30 days compared to 7 days) and a higher skuta/oil mass ratio (1.8 compared to 1.3 in this study). The reasons for these quantitative differences may be manifold, *e.g.* the use of virgin olive oil with a lower initial value of total phenols, expressed as gallic acid equivalents (311 mg/kg), a higher water mass fraction in the skuta (74 % compared to 65 % in this study) and the determination of total phenols with a method less specific for phenols (Folin-Ciocalteu) compared to the Fast Blue BB test. When comparing the influence of the two immersed materials on the total phenols in the oil, a statistically significant difference can only be observed after 21 days of storage. The decrease in total phenols under the influence of skuta stopped after 14 days of storage, while it continued until the 21st day under the influence of tofu (Table 1). This almost identical effect of the two materials in the first two weeks could be the result of a combination of at least three factors: total protein content, differences in protein properties and degree of water-to-oil migration. Considering the high affinity of the phenols in virgin olive oil to the water phase (*34*), their migration from the oil to the water droplets is very likely. The similar effect of skuta and tofu on the phenols in the oil coincides with the increase in water content in the oil, which did not differ significantly between these two materials during the mentioned period. According to the manufacturers’ nutrition data labels, the mass fraction of protein in tofu was higher (12 %) than in skuta (8.6 %). Assuming that the proteins in both matrices are the main components that can bind phenols from virgin olive oil, a greater effect would be expected for tofu. However, this does not have to be the case, as Rawel *et al.* (*35*) pointed out that the interactions between phenolic substances and proteins are significantly influenced by the amino acid composition as well as the secondary and tertiary structure of the protein molecules. Therefore, the additional decrease in the mass fraction of phenols in the oil with the addition of tofu after 21 days of storage, which was not observed in the sample with the addition of skuta, could be primarily due to a higher mass fraction of water released from the tofu during the last week of storage. Furthermore, in accordance with the conclusions of Klisović *et al.* (*4*), it cannot be excluded with certainty that other components of the food matrix (*e.g.* carbohydrates) interact with the phenolic substances in virgin olive oil. According to the manufacturers' nutritional declarations, skuta and tofu had similar average values for total carbohydrates per 100 g of product (3.5 and 3.7 g, respectively), but with significant qualitative differences – in skuta it was entirely sugar (lactose), while simple sugars in tofu accounted for only 13 % of total carbohydrates.

**Fig. 1:**
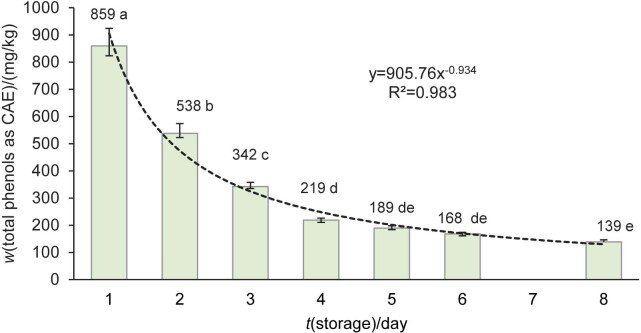
Mass fraction of total phenols expressed as caffeic acid equivalents (CAE) in virgin olive oil used as medium for the preservation of tofu during a seven-day storage at (6±2) °C (*w*(oil)=44 %). The results are given as mean value±standard deviation, *N*=4 for the control (1^st^ day) and *N*=6 for the addition of tofu (3 jars determined twice) (from 2^nd^ to 8^th^ day). Mean values labelled with different letters are statistically different (repeated measures ANOVA, Tukey’s test for unequal *N*, p<0.05)

In order to gain an insight into the dynamics of the decrease in total phenols in the oil during the first week, the storage time of the oil with tofu was reduced to 7 days and the changes from day to day were observed. In addition, the storage temperature was increased from (2±2) to (6±2) °C to avoid solidification of the oil. The decrease under the described conditions was not linear, but has the form of a rational function with a coefficient of determination R^2^>0.98 (Fig. 1), which indicates that such a regression model is highly representative. After one day of storage, the mass fraction of phenols decreased by 37 %. Since the loss of phenols due to their antioxidant activity in such a short period is unlikely, this rather indicates a very fast interaction with tofu proteins. In support of this, it can be pointed out that Quintero-Flórez *et al.* (*36*) reported a rapid reaction (within one minute) between extra virgin olive oil phenols and salivary protein mucin in aqueous solution. The large difference in the reduction of total phenols between the seventh day of the first part of the experiment (by 53 %) and the seventh day of the second part of the experiment (by 84 %) can be attributed, at least in part, to the fact that the material was kept at a lower temperature in the first part. The lower temperature led to a partial change in the aggregate state of the oil from liquid to solid, which most likely slowed the migration of phenolic substances from more distant oil layers towards the surface of the tofu pieces.

**Fig. 2:**
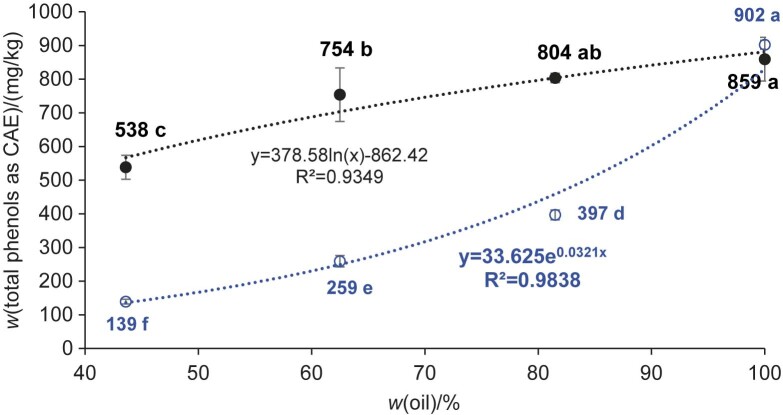
Scatter plot of the mass fraction of total phenols in virgin olive oil expressed as caffeic acid equivalents (CAE) in relation to mass fraction of oil in tofu/oil mixture on the 2^nd^ day (black dots) and 8^th^ day (blue circles) during storage at (6±2) °C. The results are given as mean value±standard deviation, *N*=6 for samples (3 jars with *w*(oil)=44, 63 and 82 % determined twice) and *N*=4 for the control (100 % oil). Mean values labelled with different letters are statistically different (repeated measures ANOVA, Tukey’s test for unequal *N*, p<0.05)

In addition, the influence of different degrees of filling the jars with tofu, expressed as the mass fraction of oil in the tofu/oil mixture, was monitored. Fig. 2 shows that on day 2 of the storage (*i.e.* after one day of contact between tofu and oil), the decrease in total phenols compared to the control was statistically significant with *w*(oil)=44 and 63 % in the tofu/oil mixture, but not with *w*(oil)=82 %, *i.e*. with the lowest filling of the jars with tofu. Nevertheless, there was a strong negative linear relationship between the mass fraction of oil in the tofu/oil mixture and the phenolic content of the oil. It is clear that with such a short contact with the surface of the immersed material, mainly phenols from the oil layers in the immediate vicinity could react. The number of such close layers of oil increases with the proportion of tofu, so that the strong negative linear relationship after one day of storage is understandable. On day 8 of storage (*i.e.* after 7 days of contact between tofu and oil), all samples, regardless of the amount of tofu filled in the jars, differed significantly in terms of total phenolic content, with the lowest decrease occurring at 82 % and the largest at 44 % oil in the mixture, as expected. After 7 days of storage, the negative linear relationship between the mass fraction of oil in the tofu/oil mixture and the mass fraction of phenols in the oil was still significant (r>-0.93). However, a comparison of the coefficients of determination (R^2^_linear_>0.86; R^2^_exponential_>0.98) shows that after 7 days of storage, the exponential regression better describes the relationship between the mass ratio of tofu/oil and the phenol content in the oil. The movement of molecules from more distant to closer oil layers due to the concentration gradient, which takes longer at lower values due to the longer path length, is an additional factor influencing the dynamics of changes in the total phenols of virgin olive oil used as a preservation medium. Such a situation can occur during occasional consumption, when pieces of food are removed from the jar several times, gradually reducing the food/oil mass ratio.

## CONCLUSIONS

A sharp decrease in total phenols in virgin olive oil during seven days of cold storage in contact with two protein-rich food matrices (skuta cheese and tofu) suggests a combination of causes, such as water-to-oil migration and phenol-protein interactions. The representativeness of the rational function for the decrease in total phenols during the first seven days of storage of tofu in oil suggests a very rapid interaction with tofu proteins. The results of this study contribute to the knowledge of the interaction dynamics between phenols and proteins and emphasise the need for further studies on traditional or newly used protein-rich foods preserved in direct contact with virgin olive oil. However, the preservative effect of virgin olive oil in terms of microbiological spoilage is not confirmed.
